# Exosomal proteins as potential markers of tumor diagnosis

**DOI:** 10.1186/s13045-017-0542-8

**Published:** 2017-12-27

**Authors:** Aichun Li, Tianbao Zhang, Min Zheng, Yanning Liu, Zhi Chen

**Affiliations:** State Key Laboratory for Diagnosis and Treatment of Infectious Diseases, The First Affiliated Hospital, College of Medicine, Zhejiang University, Collaborative Innovation Center for Diagnosis and Treatment of Infectious Diseases, 79# Qingchun Road, 6A-17, Hangzhou, 310003 China

**Keywords:** Exosomes, Isolation, Detection, Exosomal proteins, Cancer biomarker

## Abstract

Liquid biopsy especially that of exosomes carries tumor-specific molecules and provides useful information during tumor development and progression in “real time.” Exosomes are membrane-encapsulated vesicles, constantly released by multiple cells, including cancer cells, in large quantities, and are widely present in body fluids. Tumor exosomes can remodel a tumor-supportive microenvironment via cross-talk with target cells. Recent research has mainly focused on exosomal miRNAs and to a small degree on proteins. However, detecting the genome output (active proteins such as phosphoproteins) can provide more direct information about disease progression, such as in the early discovery and monitoring of cancers. This review highlights the unique features of exosomal proteins over traditional serological markers and summarizes their recent use in cancer diagnosis and prognosis. Furthermore, we describe the general protocols of research on exosome proteomics with an emphasis on their clinical use.

## Background

Traditional tissue biopsy might fail to represent tumoral heterogeneity and initiate therapy in time. In contrast, liquid biopsy, including that of circulating tumor cells (CTCs), cell-free DNA (cfDNA), and exosomes, provides comprehensive information about tumors in a non-invasive manner. Additionally, liquid biopsy can be repeatedly used to monitor cancer progress or effective treatment [1]. Liquid biopsy is an invaluable cancer biomarker reservoir.

Exosomal proteins possess unique features over traditional serological markers. First, exosomal proteins have a higher sensitivity compared with proteins directly detected in blood. Nuclear transcription factor, X-box-binding protein 1 (NFX1), and cGMP-dependent protein kinase 1 (PKG1) are only detected in plasma exosomes [2]. Specific proteins secreted by cancer cells are diluted or combined with other substances like protein-bound PSA in blood which may affect the test results, while there are over 10^9^/ml exosomes in human blood and individual exosomes typically expose 10–100 surface antigens [3, 4]. Second, exosomal proteins have a higher specificity over secretory proteins. Glypican-1 (GPC1) which was demonstrated specifically enriched on cancer cell-derived exosomes and showed great specificity over CA-199 or serum-free GPC1 (100% vs 79.49% vs 82.14%) in distinguishing non-cancer subjects from pancreas cancer patients [5]. Third, exosomal proteins are highly stable. Exosomal proteins are protected from external proteases and other enzymes by the lipid bilayer, and phosphorylation proteins can be separated from exosomal samples frozen for 5 years [2]. Tumor is extremely heterogeneous, so the chance to identify a single diagnostic biomarker is likely rare. Exosomes may act as a combination of panel candidate markers including oncogenic protein, mutation DNA, and RNA.

There are currently studies that describe the mechanism of exosomal proteins in regulating tumor progress and summarize their feasibility as tumor markers [6]. However, an understanding of how to realize the clinical application of exosomal proteins in tumor diagnosis and prognosis evaluation is still lacking. This review highlights the specific exosomal proteins in various solid tumors and compares the different exosome isolation methods and means of protein analysis in clinical application.

### Fundamental characteristics and physiological functions of exosomes

**Fig. 1:**
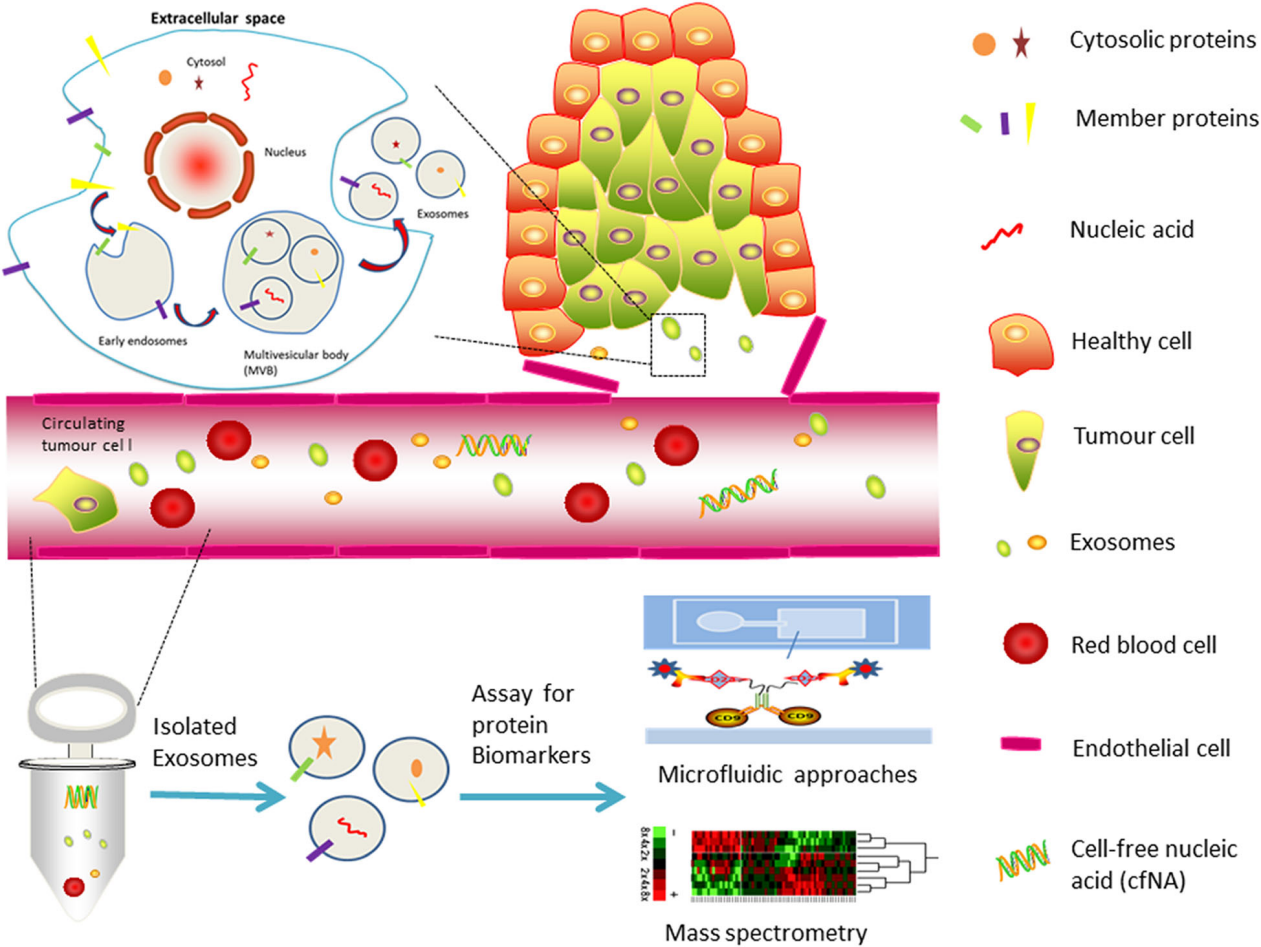
Schematic representation of exosome biogenesis, release, and isolation from the blood. Endocytosis at the plasma membrane forms the early endosome. The inward budding of the membrane of late endosomes and subsequent pinching off of the membrane creates the exosomes, called multivesicular bodies (MVBs). Upon the fusion of MVBs with the plasma membrane, exosomes are released into the extracellular milieu, which enriched in nucleic acid and proteins. Exosomes, cell-free nucleic acid (cfNA), circulating tumor cell can be found and extracted from a blood sample. Improvement in mass spectrometry-based proteomic tools and microfluidic approaches, which coupled with improved purification schemes for exosomes, has allowed more in-depth proteome analyses. The potential of exosomal protein profiles for use as diagnostic biomarkers of disease through a non-invasive blood test

Exosomes are nano-sized (40–100 nm) vesicles that are produced from multivesicular bodies (MVBs) carrying classic exosomal markers, such as CD9, CD81, and CD63 [7] (Fig. 1). There is agreement that MVBs have two distinct fates in all cells, either fusing with the plasma membrane to release exosomes into the extracellular milieu or fusing with the lysosome where their cargo is digested [8]. High levels of ceramide lipid family proteins appear to help in exosome secretion [9]. Exosomes are naturally released from normal cells and cancer cells found in the blood as well as in urine, ascites, and saliva [10–13]. Exosome cargo is assembled by enzymes, metabolites, proteins, lipids, and nucleic acids, reflecting the cell origin and the organism’s physiological conditions and cancer progression [14–19].

Exosomes were once thought to restore the dynamic and homeostatic cellular homeostasis conditions by discarding excess or harmful molecules from cells, such as by eliminating transfer receptors during reticulocyte maturation and removing harmful DNA from the cytoplasm to avoid the senescence or apoptosis of normal human cells [20–22]. In recent years, exosomes have been attributed to multiple biological functions by cell-to-cell communication through direct membrane fusion with the plasma membrane, endosomal membrane, endocytic pathways, or ligand-receptor interactions [23, 24]. Exosomes have been demonstrated to play a vital role in multiple cellular processes, such as inflammation, immune regulation, tissue regeneration, senescence, and cancer [25–28]. However, in this review, we will emphasize the role of exosomes in cancer.

### Exosomes’ functions in cancer

Tumor-derived exosomes exchange oncogenic factors with nearby, even distant, cells including cancer-associated fibroblasts, vascular endothelial cells, and immune cells to establish favorable conditions for cancerous growth and metastasis [29]. In physiological states, endogenous RNA is shielded by RNA-binding proteins [30]. However, breast cancer cells can stimulate stromal fibroblasts to produce stromal exosomes, which contain unshielded endogenous RNA (RN7SL1), via triggering NOTCH-MYC pathways. Delivering unshielded RN7SL1 to breast cancer cells facilitates cancer growth, metastasis, and therapy resistance through retinoic acid-inducible gene I (RIG-I) signaling. In addition, more unshielded RN7SL1 was detected in exosomes from patient serum than in normal controls [31]. The level of miR-23a was significantly increased in hypoxic lung cancer-secreted exosomes compared to normoxic parental cells. Exosomal miR-23a promoted angiogenesis by accumulation of hypoxia-inducible factor-1α (HIF-1α) and increased vascular leakiness by inhibiting tight junction protein zonula occludens-1 (ZO-1). Furthermore, the elevated serum levels of lung cancer-derived exosomal miR-23a highlight its potential clinical relevance and prognostic value [32]. Tumor exosomes are not only essential to directing cancer cell migration but also help to form a pre-metastatic niche before site-specific metastasis [33–36]. Tumor exosomes possess both anti- and pro-tumorigenic potentialities in the body. In chronic lymphocytic leukemia (CLL), non-coding RNAs from tumor exosomes can activate monocytes by activating TLR signaling. The activated monocytes release many cytokines, such as interleukin-6, C–C motif chemokine ligand 4 (CCL4), and CCL2, and express the programmed-death ligand 1 (PD-L1), which remodels the tumor-supportive microenvironment [37]. Tumor-derived exosomes carry and deliver tumor antigens to dendritic cells, inducing potent CD8^+^ T cell-dependent antitumor effects in animal experiments [38]. Together, these studies suggest that tumor-derived exosomes play an important role in promoting both primary tumor growth and metastatic spread.

### Technology for exosome isolation and identification and analysis of their proteins

Before launching downstream proteomic studies or functional assays, it is necessary to concentrate and characterize the exosomes in body fluids. In this section, we will summarize commonly used isolation and detection methods as well as general protocols of exosomal protein research.

### Methods for isolation exosomes

#### Ultracentrifugation (UC)

Ultracentrifugation is the gold standard for exosome isolation, accounting for over 56% users in exosome research [39]. Although ultracentrifugation can produce large amounts of exosomes with great purity, it is unsuitable for clinical diagnosis due to its low throughput which can only detect six samples simultaneously and the repeatability is poor making the study-to-study comparability questionable. Ultracentrifugation is often contained by protein aggregates, and viruses combined with density gradient centrifugation (DGC) can obtain a higher purity of exosomes, but DGC is time-consuming (centrifugation for 16–90 h) which hampers the use in a clinical setting [40]. Currently, based on ultracentrifugation method, glypican 1, PSA has been respectively identified as early diagnostic biomarkers in pancreatic cancer and prostate cancer in blood exosomes [5, 41].

#### Size exclusion chromatography (SEC)

Size exclusion chromatography is a gravity flow-based technique to separate exosomes from other vesicles with different sizes [42]. This method has the advantage of maintaining the structural integrity and biological activity of exosomes [43]. In addition, SEC is fast, 10 to 20 min per sample, and relatively inexpensive which makes SEC clinically applicable.

#### Polymer precipitation

Isolation exosomes based on polymer precipitation is simple and easy to use and require no specialized equipment and is comparable with both low- and high-sample volumes [44]. However, additional steps are required to remove lipoproteins and polymer using a Sephadex G-25 column to improve exosome separation purity [45]. Serum exosomal carcinoembryonic antigen (CEA) exhibited a better sensitivity and specificity than serum CEA using ExoQuick™ [46].

#### Immunocapture assays

Immunocapture assays can isolate specific subpopulations of exosomes using beads, plate-coated monoclonal antibodies target specific ligand expressed on exosomal surface such as proteins or phosphatidylserine (PS) [47, 48]. Commercial kits like Magcapture™ Exosome isolation kit PS and CD63 Dynabeads® beads are based on this principle [49]. Immunocapture assays have the advantages of being high throughput, rapid (4–12 h), easy to use, and compatible with routine bench equipment and therefore are clinically applicable. Recently, the level of exosomal PS in circulation was confirmed as a hopeful marker for early diagnosis of malignancies by using sensitive ELISA [50].

#### Microfluidic technologies

Although the application of microfluidic technology in exosome isolation is still in its infancy, the small sample size (5–100 μl), high purity, high sensitivity, and short operation time (1.5 h) have made microfluidic technology hold good clinical application prospect [51]. But repeatability and consistency remain one of the major challenges. Exosomes are isolated by two types of antibodies and are detected by photosensitizer beads, CD9, and CD147 double-positive exosomes that are enriched in serum from colorectal cancer patients [52].

### Strategies for exosome identification and exosomal protein analysis

It is critically important to assess the recovery and purity of isolated exosomes for proteomic experiments or functional investigations. A particle to protein ratio of 3 × 10^10^ or greater has been considered to be high purity [53]. Electron microscopy (EM) can be performed as the gold standard method to check the presence of exosomes with a cup-shaped morphology and even to check the exosomal immunophenotype by immuno-electron microscopy [3]. Nanoparticle tracking analysis (NTA) can be used to determine the concentration and size of exosomes [54]. Exosome concentrations probably range between 10^7^ and 10^9^/mL plasma in physiological conditions [3]. ELISA and Western blots are used to discover established exosomal markers, such as CD9, CD81, and CD63. The “exclusion markers,” such as calnexin or the endoplasmic reticulum heat shock protein (GP96), which are unexpected in exosomes, can be detected by Western blot [55].

Highly abundant proteins in the samples were removed using the commercial depletion kit before exosome enrichment, and isolated exosomes were further verified by morphology tests and surface biomarker analysis [56]. Indeed, these proteins are routinely removed before downstream proteomic analysis. In recent literature, the use of mass spectrometry or Extracellular Vesicle (EV) Arrays for proteome analysis is becoming extremely popular for discovering disease-specific proteins [57]. The EV Array is a sandwich ELISA-based method that explores multiple membrane-associated proteins simultaneously [58].

First, we should make sure that the disease-specific proteins are novel by comparing them with the available data in EVpedia, Exocarta, and Vesiclepedia [59–61]. Second, we need to verify these disease-specific proteins in a larger cohort. ELISA and Western blot are widely utilized methods to characterize the presence of a particular protein in exosomes. ELISA captures exosomes by targeting conservative exosomal proteins and quantitatively assesses the tumor-associated proteins. Western blot can identify both the cytosolic proteins and membrane proteins. The immuno-gold-labeling protocol, involving incubating exosomes with antibody-coated gold particles (4–40 nm), is applied to reveal the presence of tumor-specific membrane proteins under EM [62]. Because of the number of surface antigens in the exosomes (< 100) and the size (< 300–500 nm), which is below the detection limit of the conventional flow cytometers, exosomes need to bind to beads and fluorescence-labeled antibodies to evaluate their surface phenotype by most flow cytometers [4]. Recently, researchers developed high-resolution flow cytometry, which allows quantitative and multiparameter qualitative analysis of nano-sized vesicles (100–200 nm) [63, 64]. The microfluidic device can simultaneously isolate and identify exosome surface proteins without purity processing, which may provide a rapid and high-throughput platform in translational medicine [65]. However, not every research group has this device. In fact, exosome concentration is routinely detected by NTA. Finally, we need to set a cutoff value of exosomal proteins to test and verify their potential clinical relevance in another cohort. Additionally, functional assays are needed to better understand the biological mechanisms of the exosomal surface proteins in general.

### The application of exosomal proteins in tumor diagnosis

Exosomes have gained great attention due to their function in shuttling specific tumor markers in solid tumors. The concentration of exosomal proteins is higher in cancer patients compared with tumor-free individuals. In addition, tumor exosomes contain plenty of cancer biological information [35]. With the development of both proteomic technologies and analytical means for exosomal proteins, the number of papers about exosomal proteins is rapidly increasing. In this section, we highlight the clinical study of exosomal proteins in the early detection and diagnosis of cancer (Table 1).

**Table 1:** Exosomal proteins as potential diagnostic markers in various tumors

Exosomal proteins	Tumor	Body fluid	Isolation method	Detection method	Year	Ref
NY-ESO-1	Lung	Plasma	Extracellular Vesicle Array	Extracellular Vesicle Array	2016	[66]
PKG1, RALGAPA2, NFX1, TJP2	Breast	Plasma	Ultracentrifugation	Parallel reaction monitoring (PRM)	2017	[2]
Her2	Breast	Plasma	Microfluidic chip	Microfluidic chip	2017	[70]
Glypican-1	Breast	Serum	Ultracentrifugation and FACS	FACS	2016	[5]
Glypican-1	Pancreatic	Serum	Ultracentrifugation and FACS	Flow-cytometry and ELISA	2016	[5]
Glypican-1	Colorectal	Plasma	Immunocapture assays	Flow-cytometry	2017	[76]
CEA	Colorectal	Serum	Polymer precipitation	ELISA	2017	[46]
AMPN VNN1, PIGR	Cholangiocarcinoma	Serum	Ultracentrifugation	Western blot	2016	[77]
PSA	Prostate	Plasma	Filtration and ultracentrifugation	ELISA and nanoscale flow-cytometry	2017	[41]
GGT1	Prostate	Serum	Ultracentrifugation/DGC/SEC	Western blot and fluorescent probe	2017	[80]
CD24, EpCAM, CA-125	Ovarian	Plasma	Microfluidic ExoSearch Chip	Microfluidic ExoSearch Chip	2016	[86]

### Thoracic tumors (lung cancer, breast cancer)

Kristine et al. [57] developed an EV Array that coupled 37 antibodies targeting lung cancer-associated proteins and a panel of CD9, CD63, and CD81 antibodies to explore circulating exosomes from healthy subjects and lung cancer patients. The authors used a combined 30-marker model EV Array, which can successfully distinguish the two groups with 75.3% accuracy. Using a similar method, it was found that the exosomal protein of New York esophageal squamous cell carcinoma-1 (NY-ESO-1) maintained a significant concentration-dependent impact on inferior survival after multiple testing using the Bonferroni correction method [66]. This approach involved identifying and comprehensively comparing the proteome profiles of saliva and serum exosomes from lung cancer patients and healthy controls by liquid chromatography-tandem mass spectrometry (LC-MS/MS) [11]. Coincidently, a panel of 11 cancer-related proteins was detected in exosomes from the body fluids of lung cancer patients. These proteins may provide a diagnostic source for the early detection and diagnosis of lung cancer once they are validated. It was found that other tumor-specific proteins included plasma exosomal surface EGFR or leucine-rich alpha-2-glycoprotein (LRG1) in urinary exosomes, which were identified in lung cancer patients [67, 68].

The events of protein phosphorylation may provide clues about disease status [69]. However, few phosphoproteins in biofluids have been reported as disease markers due to their highly dynamic nature and the presence of active phosphatases in blood. Using LC-MS/MS, researchers identified more than 100 phosphoproteins in plasma exosomes that are significantly higher in breast cancer patients compared with healthy controls. In addition, they applied parallel reaction monitoring (PRM), a quantitative mass spectrometry (MS) approach, to verify four phosphoproteins: cGMP-dependent protein kinase 1 (PKG1), Ral GTPase-activating protein subunit alpha-2 (RALGAPA2), nuclear transcription factor, X-box-binding protein 1 (NFX1), and tight junction protein 2 (TJP2), which showed significant upregulation in breast cancer patients [2]. This study demonstrates that phosphoproteins in plasma exosomes can provide useful real-time information in the early detection and monitoring of cancers. It was shown that the level of plasma epithelial cell adhesion molecule (EpCAM)-positive exosomes was significantly higher in breast cancer patients compared with healthy individuals. Moreover, the level of human epidermal growth factor receptor-2 (HER2) in the plasma exosome was almost consistent with that in the tumor biopsies [70]. The exosomal HER2 in circulation could reflect molecular classification of the tumor tissues in a non-invasive way. The levels of exosomal fibronectin and developmental endothelial locus-1 (EDIL3) were significantly higher in breast cancer patients than controls and dramatically reduced after tumor resection, suggesting that they may serve as important diagnostic and prognostic markers for breast cancer patients [71, 72]. CD24 on circulating exosomes has emerged as a diagnostic biomarker of breast cancer patients [73]. Survivin levels were significantly higher in serum samples from all stages of breast cancer compared to the controls, and survivin was found in tumor tissues, suggesting that it was a part of the exosomes from tumor cells [74]. However, survivin-2B, which is a survivin alternative splice variant, is a pro-apoptotic protein that was inversely related to tumor grade in tumor tissues, which could be a good prognostic marker in this disease.

### Gastrointestinal tumors (pancreas cancer, colorectal cancer, cholangiocarcinoma)

Recent work has demonstrated that special proteins are only present on the exosomes derived from malignant cells [5]. Glypican-1 (GPC1), the cell surface proteoglycan, is overexpressed in breast and pancreatic cancers and is exclusively detected on exosomes derived from those malignant cells. Additionally, the level of GPC1+ exosomes in circulation correlates well with the patient’s clinical outcome after removal of pancreatic lesions. However, Lai and colleagues [75] reported that exosomal GPC1 is not able to distinguish pancreatic cancers from non-tumorous controls and that the levels of exosomal GPC1 were only slightly lower after resection. However, the levels of exosomal microRNAs were significantly elevated in pancreatic cancers and normalized following pancreas resection. Nevertheless, large variation among the clinical samples and poor consensus regarding the isolation and detection methods may hinder the integration of the data from different labs. During the early stage of pancreatic cancer progression to liver metastasis, the migration inhibitory factor (MIF) was markedly elevated [36]. Application of GPC1 as a diagnostic marker for colorectal cancer (CRC) is also reported. The study showed that both the percentage of GPC1+ exosomes and the GPC1 protein expression in exosomes from tumor tissues and plasma of CRC patients were significantly decreased after surgery compared to those in the peritumoral tissues and plasma of healthy individuals [76]. CD9^+^ CD147^+^ exosomes were abundant in CRC patient serum using “ExoScreen,” and the CD147 level dropped after surgery [52]. Serum exosomal CEA can predict metastatic CRC with a higher sensitivity and accuracy than serum CEA [46]. The high abundance of oncogenic proteins is present in both cholangiocarcinoma (CCA) human cell lines and CCA patient serum, which provided a basis for cholangiocarcinoma diagnosis. In addition, EGFR, Mucin-1 (MUC1), and integrin beta-4 (ITGB4), which promote tumor growth and metastasis, may be bad prognostic factors in this tumor [77]. In fact, exosome concentration is also a useful biomarker in bile to discriminate malignant common bile duct (CBD) stenoses from controls or non-malignant CBD stenoses with 100% accuracy [78].

### Urinary tumors (prostate cancer, bladder cancer)

Plasmatic PSA is widely used for prostate cancer (PCa) detection and monitoring. However, PSA testing fails to differentiate benign prostatic hypertrophy (BPH) from tumors [79]. It was found that the tumor microenvironmental acidity increased the release of exosomes and influenced PSA by prostate cancer cells. PSA^+^ exosomes in the plasma of PCa patients is fourfold greater than that of tumor-free controls [41]. Additionally, it was shown that gammaglutamyltransferase 1 (GGT1), a cell-surface enzyme, was present together with CD9 in exosomes from human serum. Serum exosomal GGT activity and GGT1 expression were significantly higher in PCa patients than in BPH, which may serve as a novel diagnostic marker to distinguish these diseases [80]. Through comparing the proteome of the urinary exosomes of PCa patients with healthy subjects, TM256 and ADIRF have shown the highest diagnostic value [81]. Twenty-nine urinary exosomal proteins have emerged as novel candidate biomarkers, especially tumor-associated calcium-signal transducer 2 (TACSTD2), which was directly quantified by ELISA in urine specimens and confirmed to have potential value for diagnosis of bladder cancer [82]. Bladder cancer exosomes contain EDIL-3/Del1 and facilitate cancer progression [83].

### Other tumors

Exosomal proteins have been suggested as novel diagnostic and prognostic indicators for a variety of cancers. Many melanoma-specific exosome proteins, such as caveolin-1, were identified in clinical samples [84]. EGFR, EGFRvIII, and CD63 were detected in serum exosomes of glioblastoma patients [85]. Multiple exosomal proteins, e.g., CD24 and claudin-4, can serve as promising biomarkers of ovarian cancer [86, 87]. In the future, these proteins will need to be further validated in heterogeneous and larger patient cohorts.

## Conclusions

The growing body of functional studies has provided strong evidence that these exo-based markers can be identified for early stage cancer detection, as well as to even predict clinical outcome. Currently, exosomes are isolated primarily by ultracentrifugation or immunocapture. The former is not highly specific and is unsuitable for clinical applications, while the latter may introduce bias and contaminations from serum/plasma proteins. Most of the clinical research on exosomal proteins is still based on the case data from a single group/hospital.

Reproducible isolation and highly sensitive identification methods are needed for research and development. Moreover, we need to standardize the methodology and technology for exosome isolation and identification in order to effectively integrate the data from different labs and enhance their feasibility in clinical application. In addition, their application still should depend on multicenter joint verification to further determine the cutoff value, sensitivity, specificity, and so on. With the improvements in technology, through a simple blood test, exosomal protein content or molecular/genetic profiles can provide preliminary diagnostic and prognostic information for cancer patients. Tissue biopsy and exosomes can be combined in the provision of personalized diagnosis and treatment.

## Abbreviations

ADIRF: Adipogenesis regulatory factor
BPH: Benign prostatic hypertrophy
CBD: Common bile duct
CCA: Cholangiocarcinoma
CCL2: C–C motif chemokine ligand 2
CEA: Carcinoembryonic antigen
CfDNA: Circulating free DNA
CRC: Colorectal cancer
CTCs: Circulating tumor cells
DGC: Density gradient centrifugation
EDIL3: Developmental endothelial locus-1
EDIL-3/Del1: EGF-like repeats/discoidin I-like domain-3
EGFR: Epidermal growth factor receptor
ELISA: Enzyme-linked immunosorbent assay
EM: Electron microscopy
EpCAM: Epithelial cell adhesion molecules
EV Array: Extracellular Vesicle Array
GGT1: Gammaglutamyltransferase 1
GPC1: Glypican-1
HER2: Human epidermal growth factor receptor-2
HIF-1α: Hypoxia-inducible factor-1α
ITGβ4: Integrin beta-4
LC-MS/MS: Liquid chromatography-tandem mass spectrometry
LRG1: Leucine-rich alpha-2-glycoprotein
MIF: Migration inhibitory factor
MUC1: Mucin-1
MVBs: Multivesicular bodies
NFX1: Nuclear transcription factor, X box-binding protein 1
NTA: Nanoparticle tracking analysis
NY-ESO-1: New York esophageal squamous cell carcinoma-1
PCa: Prostate cancer
PD-L1: Programmed death ligand 1
PKG1: cGMP-dependent protein kinase 1
PS: Phosphatidylserine
PSA: Prostate-specific antigen
RALGAPA2: Ral GTPase-activating protein subunit alpha-2
RIG-I: Retinoic acid-inducible gene I
SEC: Size exclusion chromatography
TACSTD2: Tumor-associated calcium-signal transducer 2
TJP2: Tight junction protein 2
TLR: Toll-like receptor
TM256: Transmembrane protein 256
ZO-1: Zonula occludens-1
